# Peer Review for “Checklist Approach to Developing and Implementing AI in Clinical Settings: Instrument Development Study”

**DOI:** 10.2196/69594

**Published:** 2025-02-20

**Authors:** Sai Saripalli

**Affiliations:** 1 Louisiana State University, Baton Rouge, LA, United States, 1 225-578-3202

**Keywords:** artificial intelligence, machine learning, ML, algorithm, model, analytics, AI deployment, human-AI interaction, AI integration, checklist, clinical workflow, clinical setting, literature review

*This is the peer-review report for “Checklist Approach to Developing and Implementing AI in Clinical Settings: Instrument Development Study.”*

## Round 1 Review

### General Comments

Using artificial intelligence (AI) to add social and domain-specific steps to clinical trials is innovative [1]. My only comment is whether the number of stages or the checklist changes if the shortlisted panelists change.

### Specific Comments

### Major Comments

1. I am unsure if having 38 (expert) panelists is good enough to have a robust framework.

## Abbreviations

AI: artificial intelligence
